# Idiopathic Multicentric Castleman Disease Presenting With Generalized Lymphadenopathy: A Case Report

**DOI:** 10.7759/cureus.117853

**Published:** 2026-10-13

**Authors:** Alma P Huerta Alvarado, Oliver Colis Arenas, Yesenia Ortega Flores, Marian Rodriguez Carrillo, Omar De los Santos Farrera, Verónica Y Campos Sánchez, Miguel Ángel Galindo López

**Affiliations:** 1 Internal Medicine, Centenario Hospital Miguel Hidalgo, Aguascalientes, MEX; 2 Teaching and Research, Centenario Hospital Miguel Hidalgo, Aguascalientes, MEX; 3 Radiation Oncology, Centenario Hospital Miguel Hidalgo, Aguascalientes, MEX; 4 Anatomical Pathology, Instituto Nacional de Ciencias Médicas y Nutrición Salvador Zubirán, Mexico City, MEX; 5 Medicine, Universidad Autónoma de Aguascalientes, Aguascalientes, MEX

**Keywords:** adrenal insufficiency, generalized lymphadenopathy, multicentric castleman disease, plasma cell infiltrate, rituximab therapy

## Abstract

Idiopathic multicentric Castleman disease (iMCD) is a rare non-clonal lymphoproliferative disorder characterized by systemic immune dysregulation and generalized lymphadenopathy, often mimicking infectious, autoimmune, and neoplastic diseases. We present the case of a 66-year-old woman with hypothyroidism and significant unintentional weight loss who was admitted with progressive dyspnea, generalized weakness, hypotension, diffuse lymphadenopathy, bibasal crackles, and ascites. Initial evaluation revealed cytopenias, elevated inflammatory markers, acute kidney injury, electrolyte abnormalities, hypoalbuminemia, diffuse lymphadenopathy, bilateral pleural effusions, ascites, and anasarca. Persistent hypotension, renal dysfunction, refractory hyperkalemia, and low serum cortisol levels raised clinical suspicion of adrenal insufficiency.

After multiple initially non-diagnostic lymph node biopsies, a subsequent cervical lymph node biopsy demonstrated histopathological and immunophenotypic findings consistent with iMCD of the mixed histological subtype, with negative human herpesvirus 8 testing and no histological evidence of malignancy. The diagnosis was established according to the Castleman Disease Collaborative Network criteria after evaluation for infectious, autoimmune, and neoplastic mimics. Intravenous hydrocortisone led to hemodynamic stabilization, renal recovery, correction of hyperkalemia, and improvement in platelet counts. The patient subsequently received rituximab, cyclophosphamide, and prednisone, with an initially favorable clinical response. However, her subsequent course was complicated by right femoral artery thrombosis requiring above-knee amputation and, later, shock associated with an extended-spectrum beta-lactamase-producing *Escherichia coli* infection, as well as progressive renal failure requiring hemodialysis. This case highlights the diagnostic complexity of iMCD and the importance of integrating clinical, laboratory, imaging, and histopathological findings, particularly when probable adrenal insufficiency is part of the initial presentation.

## Introduction

Castleman disease (CD) comprises a rare and heterogeneous group of non-clonal lymphoproliferative disorders characterized by distinctive alterations in lymph node architecture and systemic immune dysregulation. Histopathological changes occur along a spectrum ranging from hyaline vascular features, characterized by regressed or atrophic germinal centers and increased vascularity, to plasmacytic features, characterized by prominent plasmacytosis; mixed forms show characteristics of both patterns [1,2]. From a clinical perspective, CD is classified into unicentric disease (UCD), limited to a single lymph node region, and multicentric Castleman disease (MCD), which involves multiple lymph node regions and may be accompanied by a potentially severe systemic inflammatory syndrome [2-4].

CD is considered a rare disorder, and its epidemiology remains incompletely characterized [2]. The overall incidence of CD has been estimated at approximately 21-25 cases per million person-years, with iMCD accounting for approximately 5 cases per million person-years [2]. iMCD may occur across a wide age range. In a large multicenter cohort from China, patients with human herpesvirus 8 (HHV-8)-negative MCD had a median age at diagnosis of 47 years (range, 5-77 years), and 58.6% were male [1].

MCD may be further subdivided into HHV-8-associated MCD and HHV-8-negative forms. After excluding disorders capable of producing lymphadenopathy with Castleman-like histopathological changes, HHV-8-negative MCD that fulfills the required clinical and laboratory criteria is classified as idiopathic MCD (iMCD) [5]. iMCD is characterized by systemic inflammatory manifestations, cytopenias, and, in severe cases, potentially life-threatening organ dysfunction [3-6]. Its pathophysiology involves aberrant immune activation and cytokine dysregulation, particularly involving interleukin-6 (IL-6), which contributes to manifestations such as fever, weight loss, cytopenias, hypoalbuminemia, and elevated acute-phase reactants [3,4]. Histopathological patterns, including hyaline vascular, plasmacytic, and mixed variants, do not correlate directly or exclusively with the clinical classification and therefore must be interpreted together with the clinical and laboratory findings [3,4,7].

Diagnosis requires the integration of clinical, laboratory, imaging, and histopathological findings. According to the Castleman Disease Collaborative Network (CDCN) criteria, the diagnosis of iMCD requires both major criteria, at least two of 11 minor criteria, including at least one laboratory abnormality, and the exclusion of infectious, autoimmune, and malignant disorders that may mimic the disease [5]. Relevant mimics include viral infections, connective tissue diseases, lymphoma and other lymphoproliferative disorders, POEMS syndrome, and other conditions capable of producing Castleman-like histopathological changes [5,8]. Imaging studies are useful for establishing the multicentric distribution and extent of lymphadenopathy, whereas excisional lymph node biopsy remains essential for identifying compatible histopathological changes and evaluating alternative diagnoses [3-5,8].

The present case is unusual because iMCD manifested with severe systemic inflammation, generalized lymphadenopathy, cytopenias, renal dysfunction, anasarca, and a clinical presentation that raised suspicion for adrenal insufficiency. The latter could not be fully characterized endocrinologically because glucocorticoid treatment was required in the setting of critical illness. This combination illustrates both the diagnostic complexity of iMCD and the importance of integrating systemic manifestations with lymph node histopathology.

## Case presentation

We present the case of a 66-year-old woman with recently diagnosed hypothyroidism treated with levothyroxine at a dose of 150 μg daily and a five-month history of significant unintentional weight loss (22%). One week before admission, she developed progressive dyspnea and generalized weakness.

On admission (Day 0), the patient had a blood pressure of 84/42 mmHg, with a mean arterial pressure of 56 mmHg, a heart rate of 100 beats/min, a respiratory rate of 24 breaths/min, a temperature of 36.9 °C, and an oxygen saturation of 91% on room air. Physical examination revealed generalized non-tender lymphadenopathy, including left submandibular, left supraclavicular, and right inguinal lymph nodes. Bibasal crackles and clinical ascites were also noted. Initial laboratory studies demonstrated leukocytosis with neutrophilia, normocytic normochromic anemia, severe thrombocytopenia, elevated inflammatory markers, acute kidney injury, hyponatremia, severe hyperkalemia, and hypoalbuminemia (Table 1). Blood and urine cultures were negative. Initial management included norepinephrine, piperacillin/tazobactam, and hyperkalemia-directed therapy.

**Table 1:** Initial laboratory findings at hospital admission. MCV, mean corpuscular volume; MCH, mean corpuscular hemoglobin; BUN, blood urea nitrogen; ×10³/µL, thousands per microliter

Parameter	Result	Reference values
Leukocytes	18.3 × 10³/µL	4.5-11.0
Neutrophils	16.8 × 10³/µL	1.8-7.7
Hemoglobin	8.2 g/dL	12-15
MCV	82.4 fL	80-100
MCH	26.8 pg	27-34
Platelets	25 × 10³/µL	150-450
C-reactive protein	162 mg/L	<10
Erythrocyte sedimentation rate	140 mm/hour	0-20
BUN	75 mg/dL	7-17
Urea	160 mg/dL	15-36.4
Creatinine	2.9 mg/dL	0.52-1.04
Uric acid	10.5 mg/dL	2.5-6.2
Sodium	127 mmol/L	137-145
Potassium	7.9 mmol/L	3.5-5.1
Albumin	2 g/dL	3.5-5.0
Blood cultures	No bacterial growth	Not applicable
Urine culture	No bacterial growth	Not applicable

Between Days 1 and 3, contrast-enhanced computed tomography of the chest and abdomen revealed diffuse cervical, axillary, mediastinal, and inguinal lymphadenopathy, as well as bilateral pleural effusions, ascites, and diffuse subcutaneous edema consistent with anasarca (Figure 1). A 2-L therapeutic paracentesis was performed, and the analysis showed a serum-ascites albumin gradient of <1.1, normal cellularity, and a negative adenosine deaminase result. Serological testing for human immunodeficiency virus, hepatitis B virus, hepatitis C virus, cytomegalovirus, Epstein-Barr virus, and toxoplasmosis was negative. The autoimmune workup was also negative, making systemic lupus erythematosus, rheumatoid arthritis, and adult-onset Still’s disease unlikely.

**Figure 1:**
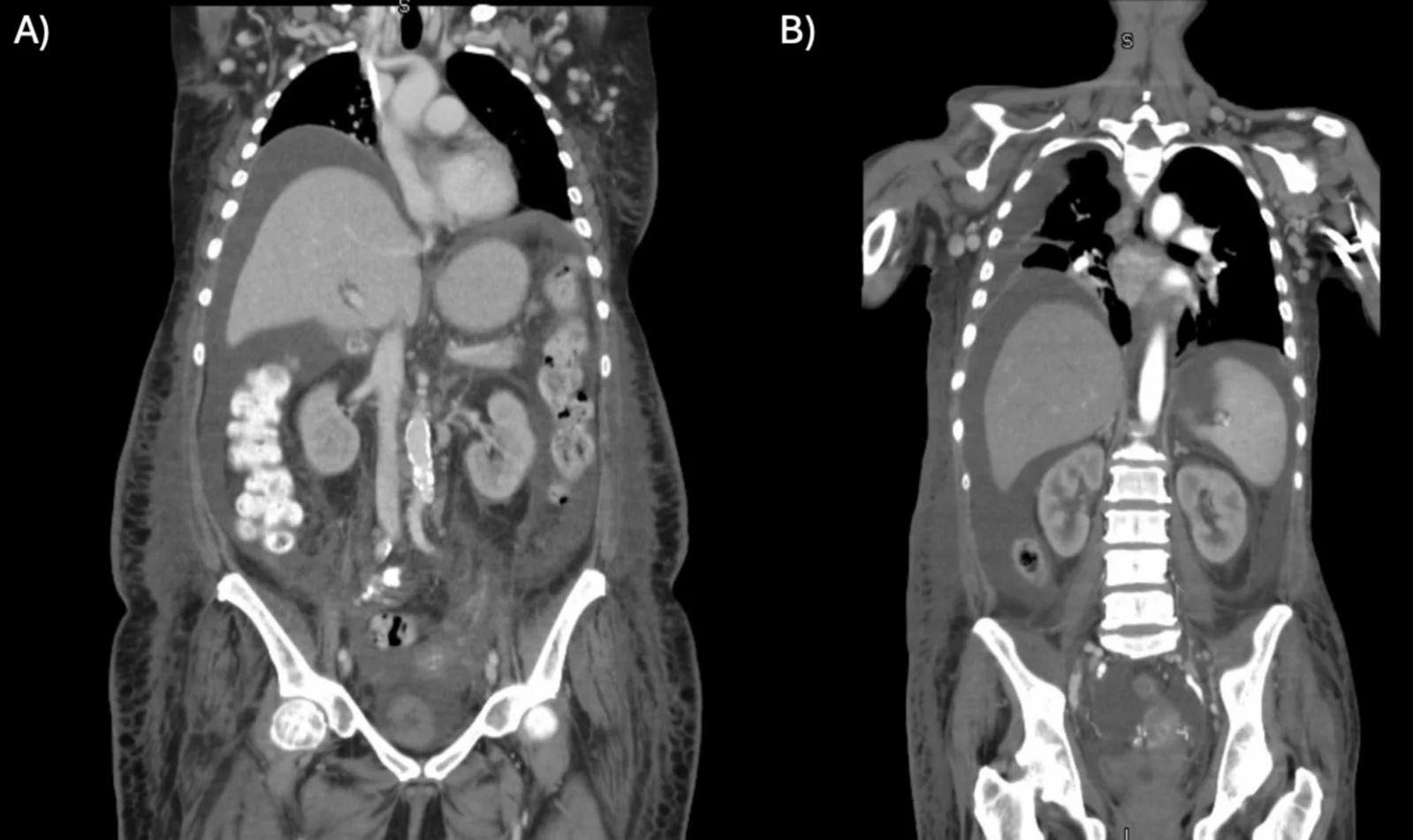
Contrast-enhanced computed tomography findings at hospital admission. (A) Coronal contrast-enhanced CT image of the abdomen and pelvis demonstrating moderate ascites, bilateral pleural effusions, and diffuse subcutaneous edema (anasarca). (B) Coronal contrast-enhanced CT image of the chest and upper abdomen demonstrating diffuse multistation lymphadenopathy involving the cervical, supraclavicular, axillary, and mediastinal lymph node stations, associated with bilateral pleural effusions.

Between Days 4 and 7, the patient developed progressive renal dysfunction and persistent hyperkalemia despite treatment, requiring the addition of vasopressin. Given the persistence of hypotension, electrolyte abnormalities, and renal dysfunction, adrenal insufficiency was clinically suspected. Low serum cortisol levels supported this diagnosis; however, complete endocrinological characterization was limited because glucocorticoid therapy was initiated early due to the patient’s critical condition. Intravenous hydrocortisone at a dose of 50 mg every six hours was started, resulting in marked clinical improvement within 72 hours, including hemodynamic stabilization, recovery of renal function, normalization of hyperkalemia, an increased platelet count, and a reduction in lymphadenopathy size.

Between Days 7 and 14, three excisional lymph node biopsies were performed; however, histopathological examination showed no evidence of malignancy or tuberculosis. During the diagnostic evaluation, a right ovarian mass associated with ascites raised concern for an underlying ovarian malignancy. Therefore, a right salpingo-oophorectomy was performed, and histopathological examination showed no evidence of malignancy. Due to persistent clinical suspicion of a lymphoproliferative disorder, a fourth excisional cervical lymph node biopsy was performed between Days 15 and 20.

Histopathological examination of the fourth biopsy demonstrated partially preserved lymph node architecture with regressive germinal centers, prominent vascular proliferation with hyalinized vessels, and interfollicular expansion by dense plasma cell infiltrates without significant cytologic atypia. No monomorphic pattern or malignant lymphoid proliferation was identified (Figure 2).

**Figure 2:**
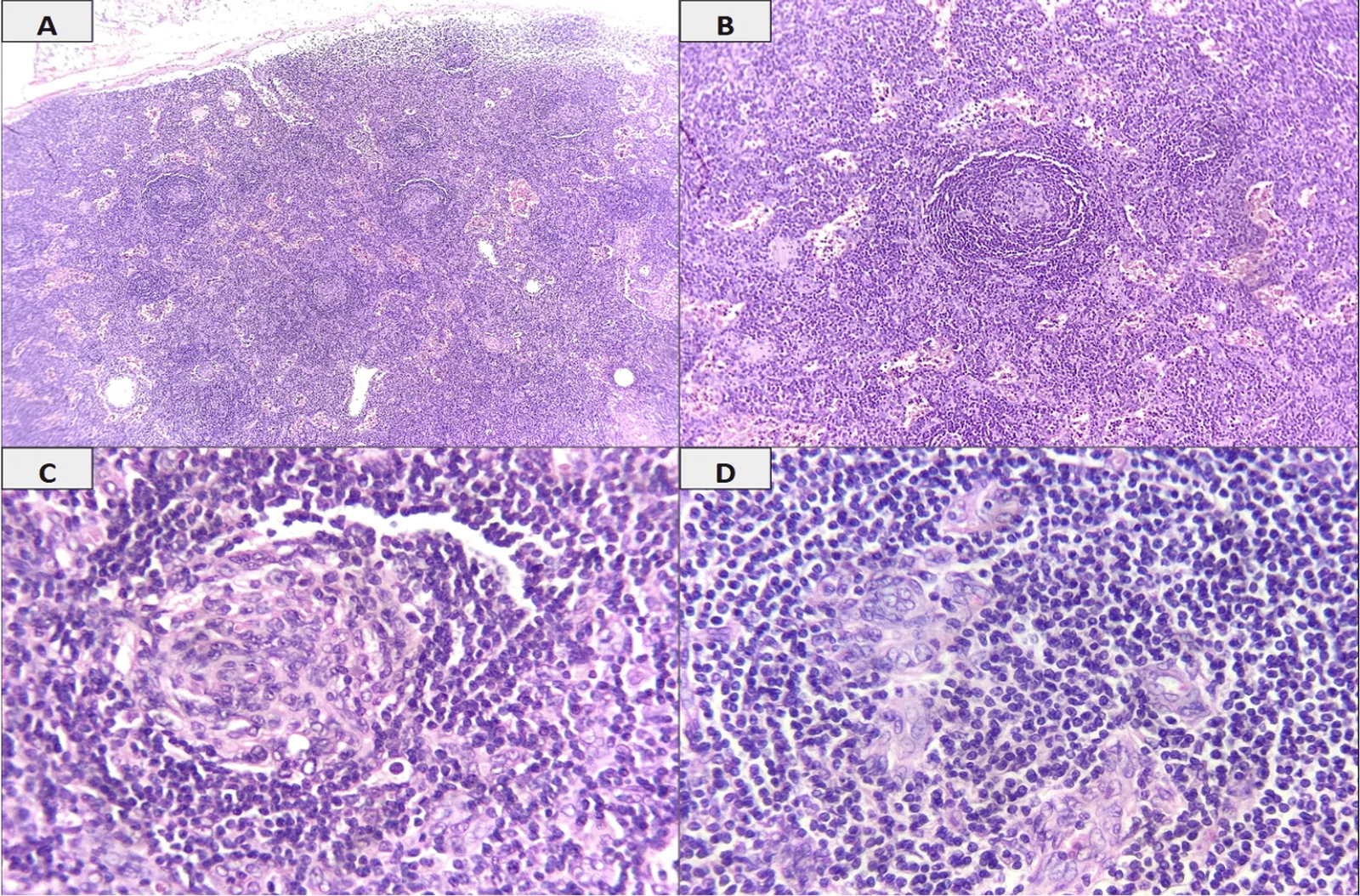
Histopathological findings of Castleman disease. (A) Hematoxylin and eosin-stained lymph node section showing multiple small lymphoid follicles with germinal centers containing blood vessels with hyalinized walls at 4× magnification. (B) Hematoxylin and eosin-stained lymph node section showing a thickened mantle zone arranged concentrically around the germinal centers at 10× magnification. (C) Hematoxylin and eosin-stained lymph node section showing a thickened mantle zone arranged concentrically around the germinal centers, as well as blood vessels with hyalinized walls, at 40× magnification. (D) Interfollicular region showing high endothelial venules and multiple blood vessels with sclerotic walls at 40× magnification.

Immunophenotyping demonstrated CD20 positivity in follicular B lymphocytes, CD138 positivity in interfollicular plasma cells, and CD5 positivity in interfollicular T lymphocytes. BCL2 expression was positive in the mantle zone, consistent with a reactive pattern. Ki-67 showed heterogeneous proliferation in germinal centers and the paracortex. Immunohistochemical staining for HHV-8 latent nuclear antigen-1 (LANA-1) was performed on lymph node tissue and yielded a negative result. Overall, the findings were compatible with iMCD, mixed histological subtype, comprising hyaline vascular and plasma cell features (Figure 3).

**Figure 3:**
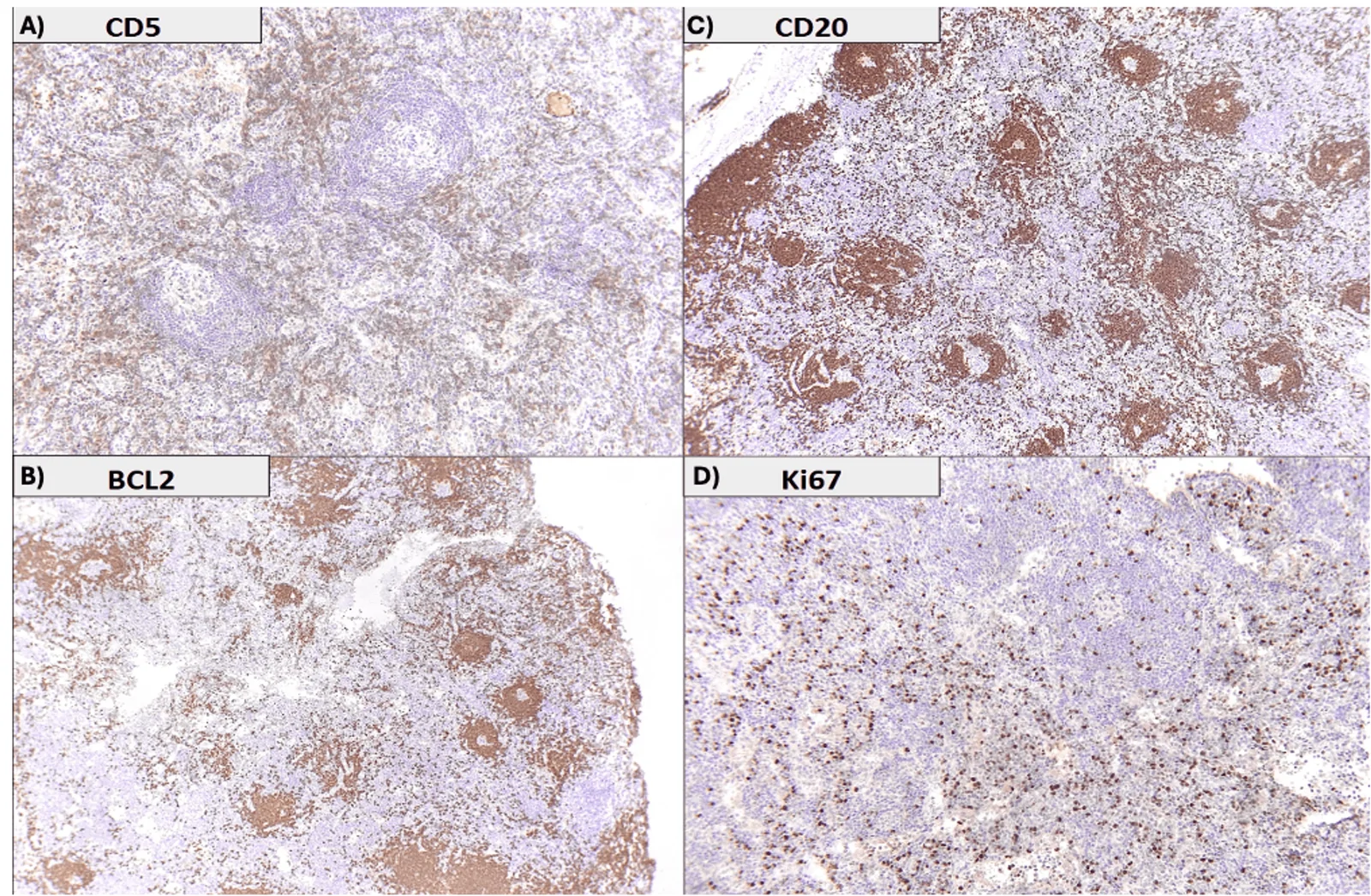
Immunohistochemistry findings. (A) CD5 is positive in the interfollicular region. (B) BCL2 is positive in the mantle zone B lymphocytes and paracortical T lymphocytes. (C) CD20 is positive in the lymphoid follicles. (D) Ki-67 shows heterogeneous staining in germinal centers and the paracortex.

Based on the Castleman Disease Collaborative Network criteria, the diagnosis of iMCD, mixed histological subtype, HHV8-negative, was established. Both major criteria were fulfilled: compatible lymph node histopathology and multicentric lymphadenopathy involving two or more lymph node stations. Several minor criteria were also present, including significant unintentional weight loss, fluid accumulation with ascites and bilateral pleural effusions, anemia, thrombocytopenia, hypoalbuminemia, renal dysfunction, and elevated acute-phase reactants. Ferritin, interleukin-6, and serum immunoglobulin levels were not available.

Alternative diagnoses were extensively excluded. Lymphoma was considered unlikely based on multiple lymph node biopsies showing no evidence of malignant lymphoid proliferation and a reactive immunohistochemical distribution. Tuberculosis was considered unlikely based on negative histopathology and normal adenosine deaminase levels in ascitic fluid. Autoimmune diseases, including systemic lupus erythematosus, rheumatoid arthritis, and adult-onset Still’s disease, were excluded based on clinical evaluation and negative serological studies. Chronic viral infections were excluded through serological testing, and imaging studies showed no evidence of occult solid neoplasms.

After the histopathological diagnosis of iMCD was established, the patient was discharged on prednisone at 1 mg/kg. She subsequently received rituximab at 375 mg/m² once weekly for six doses and cyclophosphamide at 750 mg/m² once monthly for four doses, with a favorable initial clinical response characterized by resolution of lymphadenopathy and normalization of renal function and other laboratory abnormalities. The diagnostic and therapeutic course is summarized in Figure 4.

**Figure 4:**
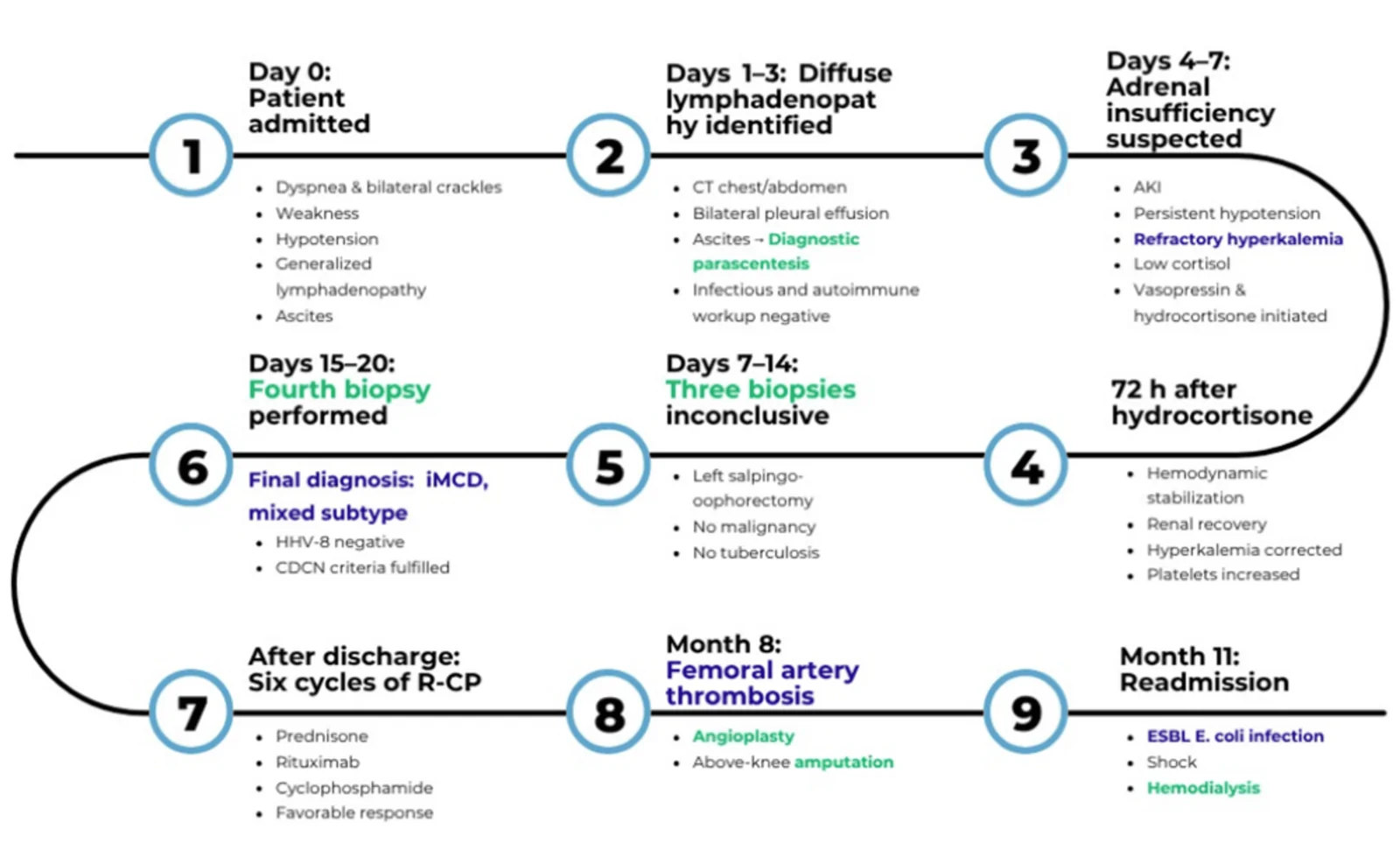
Diagnostic and therapeutic timeline of the patient with idiopathic multicentric Castleman disease (iMCD). Image created using Canva (version 1.120.0.0; Canva Pty Ltd., Sydney, Australia).

At eight months of follow-up, the patient developed right femoral artery thrombosis requiring angioplasty. Due to irreversible ischemia, a right above-knee amputation was performed.

Three months later, at month 11, she was readmitted with decreased oral intake, dyspnea on minimal exertion, generalized weakness, hypotension, and fever. Physical examination revealed signs of hypoperfusion, dehydration, and bilateral crackles. Laboratory studies showed leukocytosis with neutrophilia, elevated inflammatory markers, chronic mild hypotonic hyponatremia, moderate hyperkalemia, mild hyperchloremia, hyperphosphatemia, hypermagnesemia, and worsening renal function. Chest computed tomography demonstrated diffuse bilateral ground-glass opacities. Urinalysis was abnormal, and urine culture isolated extended-spectrum beta-lactamase (ESBL)-producing *Escherichia coli*. Meropenem treatment was initiated.

During hospitalization, the patient developed shock requiring norepinephrine and progressive renal failure, ultimately requiring renal replacement therapy with hemodialysis. The patient subsequently experienced recovery of renal function, with resolution of renal failure. She remains under outpatient follow-up.

## Discussion

MCD represents a diagnostic challenge because of its clinical heterogeneity and overlap with infectious, autoimmune, inflammatory, and lymphoproliferative disorders [3-5]. In the present case, generalized lymphadenopathy associated with severe systemic inflammation, cytopenias, acute kidney injury, ascites, pleural effusions, and anasarca initially generated a broad differential diagnosis. Multicentric forms of CD are frequently associated with constitutional symptoms and laboratory abnormalities related to immune dysregulation and increased cytokine production, particularly IL-6. The clinical presentation was characterized by severe multisystem involvement, including shock requiring vasopressor support, severe thrombocytopenia (25 × 10³/µL), renal dysfunction (creatinine 2.9 mg/dL), marked hypoalbuminemia (2 g/dL), anasarca, and bilateral pleural effusions. These findings were consistent with a severe clinical presentation of iMCD and were associated with significant organ dysfunction [3,4].

Multicentric disease can be divided into HHV-8-associated and HHV-8-negative forms, with iMCD diagnosed after fulfillment of established clinical and histopathological criteria and exclusion of alternative disorders [5]. iMCD may also present with specific clinical phenotypes, particularly the thrombocytopenia, anasarca, fever, reticulin fibrosis/renal dysfunction, and organomegaly (TAFRO) phenotype [5].

In our patient, severe thrombocytopenia (25 × 10³/µL), anasarca with ascites and bilateral pleural effusions, systemic inflammation, and acute kidney injury (creatinine 2.9 mg/dL) raised the possibility of a TAFRO phenotype. Although bone marrow examination was not performed to assess reticulin fibrosis, renal dysfunction was considered when evaluating this phenotype. The hematology team considered the lymph node histopathological findings sufficient to initiate treatment, and bone marrow evaluation was therefore not pursued. Consequently, the available information did not allow definitive confirmation or exclusion of the TAFRO phenotype. This distinction is important given the substantial clinical overlap between TAFRO and other presentations within the iMCD spectrum [5,9-12].

POEMS syndrome was also considered because of the presence of systemic disease and endocrinopathy. However, the absence of documented polyneuropathy and of evidence supporting a monoclonal plasma cell disorder made this diagnosis less likely. Careful differential evaluation is essential because of the clinical and histopathological overlap among these disorders [3-5].

Histopathological evaluation was central to establishing the diagnosis. CD encompasses a morphological spectrum that includes hyaline vascular, plasmacytic, and mixed patterns [7]. The principal findings in this patient, regressed germinal centers, concentrically arranged mantle zones, prominent vascular proliferation with hyalinized vessels, and interfollicular expansion by plasma cells, are recognizable features within this spectrum. The combination of hyaline vascular and plasmacytic characteristics supported classification as a mixed histological pattern. Importantly, histological subtype alone does not define the clinical form or severity of CD; pathological findings must be interpreted in conjunction with the multicentric distribution of lymphadenopathy and systemic manifestations [3,7,8].

The diagnosis required repeated tissue evaluation. Earlier lymph node specimens did not establish a definitive diagnosis, whereas subsequent cervical lymph node examination demonstrated the combination of regressed follicles, concentric mantle zones, vascular changes, and interfollicular plasmacytosis necessary to recognize the CD pattern. The available records do not contain sufficient pathological detail from each previous specimen to determine whether the earlier biopsies lacked characteristic areas because of sampling, showed incomplete morphological features, or were limited for another technical reason. Therefore, a specific explanation for each non-diagnostic biopsy cannot be established. Nevertheless, the clinical course illustrates the importance of repeat excisional biopsy when suspicion for a lymphoproliferative or inflammatory lymph node disorder remains high despite initially inconclusive pathological studies [8].

Similarly, the available pathological information does not document flow cytometry, immunoglobulin or light-chain restriction studies, or molecular clonality testing. The absence of malignant morphology and the reactive immunohistochemical distribution supported a non-malignant process, but it would be inappropriate to state that lymphoma was excluded through demonstrated polyclonality or *absence of monoclonality* without a specific study establishing this finding.

Another unusual aspect of the case was the clinical suspicion of adrenal insufficiency. Acute adrenal insufficiency has rarely been described in association with CD. Crump et al. reported such a presentation in 1995 [7], and subsequent reports involving TAFRO syndrome have described adrenal hemorrhage or necrosis [9-12]. These observations suggest that adrenal involvement can occur in the setting of severe systemic inflammatory disease, although the mechanism and causal relationship remain uncertain.

In our patient, suspicion of adrenal insufficiency was based on persistent hypotension requiring vasopressors, severe hyperkalemia, hyponatremia, renal dysfunction, a low serum cortisol measurement, and marked clinical improvement after administration of intravenous hydrocortisone. Nevertheless, the endocrinological evaluation was incomplete. ACTH was not measured, an ACTH stimulation test was not performed, and dedicated adrenal imaging was not obtained to investigate hemorrhage, infarction, or necrosis. Furthermore, the exact serum cortisol concentration and time of sampling were unavailable in the records reviewed. It was therefore not possible to distinguish primary from secondary adrenal insufficiency or to biochemically confirm the diagnosis according to a complete endocrine evaluation. The marked hypoalbuminemia (2 g/dL) and critical illness may also have affected the interpretation of total serum cortisol concentrations because of alterations in cortisol-binding proteins. Therefore, the reported low cortisol level should be interpreted with caution and cannot independently establish adrenal insufficiency [3,4].

For these reasons, this manifestation is more appropriately described as probable or clinically suspected adrenal insufficiency rather than definitively established adrenal insufficiency. The temporal response to hydrocortisone supports the clinical suspicion but does not establish a causal relationship between iMCD and adrenal dysfunction. In particular, unlike previously reported cases involving adrenal hemorrhage or necrosis [10-12], structural adrenal injury was not demonstrated in this patient because dedicated adrenal imaging was not performed.

Therapeutic strategies for iMCD include approaches directed against IL-6 signaling as well as other immunomodulatory or cytotoxic regimens depending on disease severity, clinical phenotype, treatment availability, and individual patient factors [3,4]. In our institution, anti-IL-6 therapies, including siltuximab and tocilizumab, were not available during the patient's treatment period. Consequently, an alternative immunomodulatory and cytotoxic regimen was selected. In this case, treatment consisted of rituximab, cyclophosphamide, and prednisone, with an initially favorable response characterized by resolution of lymphadenopathy and improvement in renal function. However, this initial response should be interpreted in the context of the subsequent complicated clinical course, including arterial thrombosis requiring above-knee amputation and a later hospitalization complicated by shock and progressive renal failure requiring hemodialysis.

Several limitations of this report should be acknowledged. Complete endocrinological characterization of the suspected adrenal insufficiency was not possible because ACTH and ACTH stimulation testing were not performed and the exact cortisol value and sampling time were unavailable. Dedicated adrenal imaging was also not performed. Bone marrow examination was not obtained, preventing complete evaluation of a possible TAFRO phenotype. IL-6, ferritin, and serum immunoglobulin measurements were unavailable. Detailed histopathological information from each of the earlier non-diagnostic lymph node specimens was incomplete, and no flow cytometry, light-chain restriction, or molecular clonality studies were documented in the available records. Finally, complete serial numerical laboratory values after initiation of therapy were unavailable, limiting the quantitative assessment of treatment response.

Despite these limitations, the present case highlights the importance of integrating clinical, laboratory, radiological, and histopathological findings in patients with generalized lymphadenopathy and multisystem inflammatory manifestations. It also demonstrates that repeat excisional lymph node biopsy may be necessary when clinical suspicion remains high despite initially inconclusive pathological evaluation.

## Conclusions

iMCD is a rare lymphoproliferative disorder characterized by multicentric lymphadenopathy and systemic immune dysregulation, with clinical manifestations that may overlap substantially with infectious, autoimmune, inflammatory, and neoplastic diseases. This case illustrates the diagnostic difficulty of iMCD in a patient presenting with generalized lymphadenopathy, severe systemic inflammation, cytopenias, anasarca, renal dysfunction, and shock. Repeated tissue evaluation ultimately demonstrated characteristic histopathological findings, emphasizing the importance of excisional lymph node biopsy and clinicopathological correlation when initial investigations are inconclusive.

The patient's presentation also raised strong clinical suspicion for adrenal insufficiency because of persistent hypotension, electrolyte abnormalities, renal dysfunction, low serum cortisol, and improvement following hydrocortisone administration. However, because ACTH measurement, stimulation testing, and dedicated adrenal imaging were not performed, adrenal insufficiency could not be completely characterized or definitively linked to iMCD. It should therefore be regarded as probable or clinically suspected adrenal insufficiency rather than a confirmed direct manifestation of Castleman disease.

Although the patient initially responded favorably to systemic treatment, with improvement in lymphadenopathy and renal function, her subsequent course was clinically significant and included right femoral artery thrombosis ultimately requiring above-knee amputation, followed by readmission with an ESBL-producing *Escherichia coli* infection, shock requiring vasopressor support, and progressive renal failure requiring hemodialysis. Thus, the complete clinical course illustrates both the initial treatment responsiveness and the substantial later morbidity that may accompany severe multisystem disease. Despite these complications, the patient subsequently recovered renal function and remains under outpatient follow-up.
